# Seroprevalence of Toxoplasmosis in Puerperal Women Treated at a Tertiary Referral Hospital

**DOI:** 10.1055/s-0043-1764495

**Published:** 2023-03-28

**Authors:** Juliana Fernandes Medeiros, Ana Cláudia Rabelo e Silva, Natália Domene Franco da Rocha, Alexia Viegas Georg, Patricia Pereira dos Santos Melli, Silvana Maria Quintana, Geraldo Duarte

**Affiliations:** 1 Faculdade de Medicina, Universidade de São Paulo, Ribeirão Preto, SP, Brazil

**Keywords:** Toxoplasmosis, Pregnancy, Vertical transmission, Health education, Prevalence, Toxoplasmose, Gravidez, Transmissão vertical, Educação em saúde, Prevalência

## Abstract

**Objective** To evaluate the seroprevalence of toxoplasmosis among puerperal women cared for at a tertiary university hospital and the level of understanding of these puerperal women about toxoplasmosis, vertical transmission, and its prophylaxis.

**Methods** For this cross-sectional study, we evaluated 225 patients using presential interviews, prenatal documentation, and electronic medical records. Data were stored using Research Electronic Data Capture (REDCap) software. Prevalence rates were estimated by the presence of reactive IgG antibodies against *Toxoplasma gondii* . Data analysis was performed using the chi-square test and calculation of the odds ratio (OR). Seroreactivity to *T. gondii* and exposure variables (age, educational level, and parity) were analyzed using a confidence interval (95%CI) and a significance level of 5% (p < 0.05).

**Results** The seropositivity rate for *T. gondii* was 40%. There was no association between seroprevalence and age. Primiparity was a protective factor against seropositivity and low education was a risk factor.

**Conclusion** Knowledge of *T. gondii* infection and its transmission forms was significantly limited, presenting a risk for acute maternal toxoplasmosis and vertical transmission of this protozoan. Increasing the education level regarding the risk of toxoplasmosis during pregnancy could reduce the rates of infection and vertical transmission of this parasite.

## Resumo

**Objetivo** Avaliar a soroprevalência de toxoplasmose entre puérperas atendidas em um hospital universitário terciário e o nível de compreensão dessas puérperas sobre toxoplasmose, transmissão vertical e sua profilaxia.

**Métodos** Para esse estudo transversal, foram avaliadas 225 pacientes utilizando entrevistas presenciais, documentação de pré-natal e prontuário eletrônico. Os dados foram armazenados usando o software Research Electronic Data Capture (REDCap). As taxas de prevalência foram estimadas pela presença de anticorpos IgG reativos contra o *Toxoplasma gondii* . A análise dos dados foi realizada por meio do teste do qui-quadrado e cálculo do odds ratio (OR). A sororreatividade ao *T. gondii* e as variáveis de exposição (idade, escolaridade e paridade) foram analisadas, utilizando-se intervalo de confiança (IC95%) e nível de significância de 5% (p < 0,05).

**Resultados** A taxa de soropositividade para *T. gondii* foi de 40%. Não houve associação entre soroprevalência e idade. A primiparidade foi fator de proteção contra a soropositividade e a baixa escolaridade foi fator de risco.

**Conclusão** O conhecimento da infecção por *T. gondii* e suas formas de transmissão foi significativamente limitado, apresentando risco para toxoplasmose materna aguda e transmissão vertical desse protozoário. Aumentar o nível de escolaridade quanto ao risco de toxoplasmose durante a gravidez pode reduzir as taxas de infecção e transmissão vertical desse parasita.

## Introduction

Toxoplasmosis is a protozoan infection that can be transmitted vertically when acquired during pregnancy. The infection of pregnant women can result in congenital infection and, consequently, fetal defects and embryonic/fetal/neonatal loss, especially if acquired in the embryonic period. 1

As most maternal toxoplasmosis cases are asymptomatic, universal serological testing is the only effective way of detecting the infection, thus enabling early vertical transmission prevention measures or therapeutic decisions. 2 3 4 Based on this context, avoiding maternal infection during pregnancy is the primary healthcare focus to control vertical transmission cases. Early therapy is also important but reflects the missed crucial prophylactic opportunity to prevent infection. 5

One of the major limitations to controlling vertical transmission of toxoplasmosis is the effective implementation of pre- and perinatal care measures, ranging from prophylaxis to avoid maternal infection to accurate diagnoses and early treatment. 5

The risk of acute maternal toxoplasmosis on the genesis of congenital malformations justifies the significant concern in this topic and requires several control measures to control the vertical transmission of *T. gondii* . These measures include epidemiological characterization, population awareness, early detection of maternal infection, and implementation of therapy.

Based on this context, the aim of this study is to evaluate the seroprevalence of toxoplasmosis among puerperal women cared for at a tertiary university hospital and the level of understanding of these puerperal women about toxoplasmosis, vertical transmission, and its prophylaxis.

## Methods

This cross-sectional study was conducted between July 2020 and August 2021 and was approved by the Research Ethics Committee of Hospital das Clínicas of the School of Medicine of Ribeirão Preto, University of São Paulo (HC-FMRPUSP) under no. 4,048,850. Postpartum women admitted to the high-risk puerperium ward of HC-FMRPUSP during the study period were enrolled if they met the inclusion criteria (all mothers in the immediate postpartum period who stayed hospitalized enough time to be interviewed, with no limit of the postpartum period, and who had toxoplasmosis exams performed) and agreed to participate in the study.

Based on previous studies 6 7 8 reporting that the mean prevalence of seronegative pregnant women to toxoplasmosis was 30% and assuming a relative error of 20% with a 95% confidence interval, 225 patients (sampling units) were necessary to detect any significant differences. The estimate was based on a mathematical equation to calculate sample size in cross-sectional studies assuming a non-absolute relative error. 9

There was no age limit for enrollment provided the underage puerperal participant's guardian acknowledged and signed both the Informed Consent (ICF) and the Informed Assent (IAF) forms. The exclusion criteria were postpartum women identified as not having psychological and/or cultural ability to understand the ICF and those isolated due to SARS-CoV-2 infection. After agreeing to participate, the women read and signed the ICF; underage participants had their guardians sign the ICF and IAF.

After consent was obtained, a questionnaire was administered to collect personal information, and an interview was conducted to collect information about the puerperal women's knowledge of the topics addressed: toxoplasmosis disease, infection transmission forms, vertical transmission, and prophylaxis for toxoplasmosis. Then, a dialogue and expository presentation about the infection were conducted, with subsequent delivery of an informative pamphlet and any other information requested by the participants about aspects of toxoplasmosis.

The data collected in interviews were complemented by an evaluation of the patients' electronic medical records at HC-FMRPUSP and at the Hygia System, maintained by the Health Service of the Health Department of Ribeirão Preto. According to the prenatal care protocols from the Brazilian Ministry of Health, the exams to identify pregnant women susceptible to *T. gondii* infection are carried out in the first trimester of pregnancy and when the results are negative, these exams must be repeated between the 28 ^th^ and 32 ^nd^ . 10 These results were retrieved from the HC-FMRPUSP and Hygia databases. When test results were not available in any of these systems (11 patients), a blood test was requested, with the patient's authorization, to detect antibodies against *T. gondii* during hospitalization in the puerperal period. Then, the data was stored and managed using the Research Electronic Data Capture (REDCap) system, 11 a tool that guarantees the security and confidentiality necessary for research information management.

The outcome variable was seronegativity to toxoplasmosis and the exposure variables were age, parity, and education level. The participants' knowledge of toxoplasmosis and its vertical transmission and prophylaxis was assessed.

SAS version 9.4 was used to analyze data frequency, consistency, and distribution and to calculate the mean and standard deviation (SD) of seronegativity to *T. gondii* and the exposure variables (age, education, and parity). Prevalence rates were estimated by the presence of reactive IgG antibodies to *T. gondii* . Variables associated with the toxoplasmosis outcome were analyzed by the chi-square test. The odds ratio (OR) was used to measure the association between seroreactivity to *T. gondii* and the variables were analyzed at a 95% confidence interval (95%CI) and 5% significance level (p < 0.05).

## Results

**Fig. 1:**
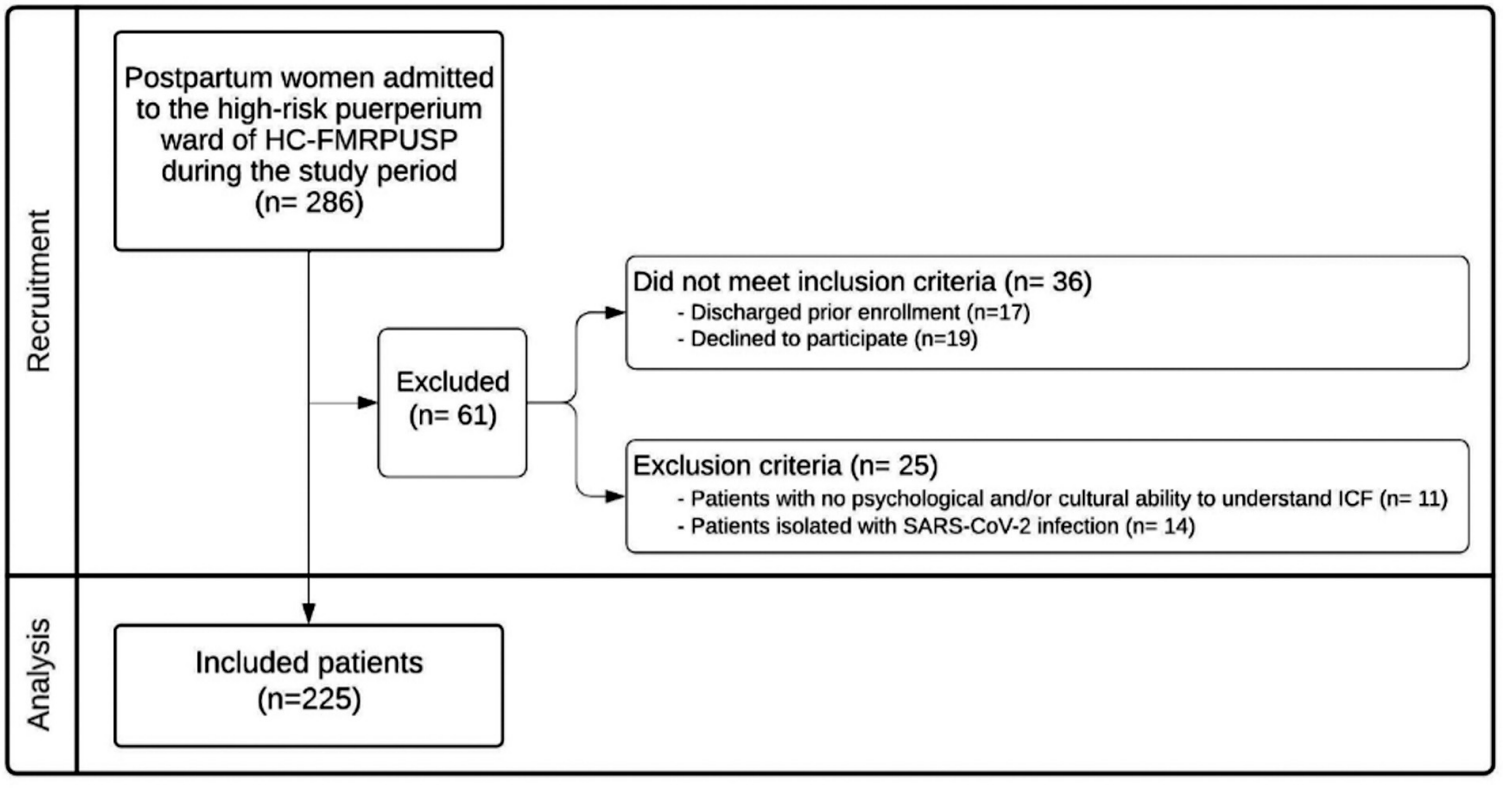
Flowchart of patient's inclusion

Of the 286 postpartum women initially included in the study, 61 were excluded for not meeting the inclusion criteria or due to the exclusion criteria, resulting in a total of 225 patients evaluated (sampling number) ( Figure 1 ).

Table 1 summarizes clinical and demographic information of the postpartum women, characterizing the patient sample studied. The mean age of the patient cohort was 29 years (SD = 7), with 34.4% of the women being primiparous and 27.6% of women having elementary education (up to 9 years of formal education) or less.

**Table 1:** Characterization of the parturients participating in the study

Parameters	n (%)
Age	
< 18 years	8 (3.5)
18 to 25 years	69 (30.7)
26 to 32 years	81 (36.0)
> 32 years	67 (29.8)
Pregnancy history	
Primiparous	77 (34.3)
Two pregnancies	57 (25.3)
Three pregnancies	43 (19.1)
Multiparous	48 (21.3)
Education level	
Elementary school or lower (≤ 9 years)	62 (27.6)
High school (9-12 years)	137 (60.9)
Higher education (≥1 year)	26 (11.5)
Total	225

The seroprevalence of toxoplasmosis in the studied group was 40% (90 of 225 patients). No association was detected between seroprevalence and the age of the pregnant women (p = 0.0790). Pregnant women with elementary education or less had statistically higher rates of seroprevalence against toxoplasmosis (p = 0.0028). Also, a positive association was observed between higher seroprevalence rates and greater parity in this sample (p = 0.0215) ( Table 2 ).

**Table 2:** Relation between the exposure variables (age, educational level, parity) and the seroprevalence of toxoplasmosis

Parameters	Seropositive	Seronegative	*p-value*
	n (%)	n (%)	
Age			
< 18 years	4 (4.4)	4 (3.0)	*0.0790*
18 - 25 years	23 (25.6)	46 (34.1)	
26 - 32 years	28 (31.1)	53 (39.3)	
> 32 years	35 (38.9)	32 (23.7)	
Educational level			
Elementary education or lower	32 (35.6)	30 (22.2)	*0.0028*
High school	51 (56.7)	86 (63.7)	
Higher education	7 (7.8)	19 (14.1)	
Parity			
Primiparous	20 (22.2)	57 (42.2)	*0.0215*
Two pregnancies	27 (30.0)	30 (22.2)	
Three pregnancies	21 (23.3)	22 (16.3)	
Multiparous	22 (24.4)	26 (19.3)	

Regarding risk ratio, completing only elementary school or less education was a risk factor for seropositivity against toxoplasmosis (OR = 2.894; 95%CI = 1.065–7.862). In turn, primiparity was a protective factor against seropositivity (OR = 0.415; 95%CI = 0.193–0.889). Regarding patients' knowledge of the disease, 32.4% said they had never heard of toxoplasmosis. Besides, almost half (43.3%) of seropositive patients belonged to this group that was unaware of this disease. Out of those who reported having heard of toxoplasmosis, 53.3% could not explain what the disease was ( Table 3 ).

**Table 3:** Patients' knowledge about toxoplasmosis

Response	“Have you heard about toxoplasmosis?”	“If yes, do you know what toxoplasmosis is?”		Seropositive n (%)	Seronegative n (%)
	n (%)		n (%)		
Yes	152 (67.6)	Yes	71 (46.7)	20 (22.2)	51 (37.8)
		No	81 (53.3)	31 (34.1)	50 (37.0)
No	73 (32.4)			39 (43.3)	34 (25.2)
Total	225 (100.0)		152	90	135

When asked about how toxoplasmosis is transmitted, 54.2% (122/225) of the patients reported not knowing the transmission method and expressed no opinion. The responses of the 103 pregnant women (45.8%) who reported knowing how toxoplasmosis was acquired are shown in table 4 . The most cited form of transmission was contact with domestic animals (25.7%), while only two patients (0.9%) mentioned vertical transmission. Also, some unusual forms such as “birds/rats” (1.8%), sexual transmission (1.3%), “saliva or sneezing” (0.9%), and “domestic animal bites” (0.9%) were also mentioned as possible transmission routes. Percentages were not calculated since each patient recorded more than one response regarding *T. gondii* infection transmission routes.

**Table 4:** Patients' knowledge of toxoplasmosis transmission

“How do you think toxoplasmosis is transmitted?”	Elementary education or lower n answers	High school n answers	Higher education n answers
Contact with domestic animals (58)	13	34	11
Contact with animal feces (40)	5	27	8
Consumption of poorly washed food (30)	4	19	7
Consumption of raw or undercooked meat (27)	5	15	7
From mother to fetus (2)	0	1	1
Contact with other animals: birds/rats (4)	2	1	1
Sexual intercourse (3)	2	1	0
Saliva or sneezing (2)	1	1	0
Domestic animal bite	1	1	0
Do not know	70	33	0

## Discussion

The seropositivity rate of antibodies against *T. gondii* in the analyzed group was 40%, which demonstrates a potential susceptibility in 60% of childbearing-age women who have never had contact with the protozoan but who could still be infected in future pregnancies. These data contrast with the high seropositivity rate (59%) reported in a similar population in 2004, 5 which presented a potential susceptibility and risk of vertical transmission in 41% of women at that time. Improved sanitary standards in Ribeirão Preto and the region resulted in a greater number of women reaching reproductive age without previous contact with the protozoan. This lack of prior exposure necessitates specific and extensive prophylactic guidance in prenatal care.

The seroprevalence in the present study (40%) is one of the lowest recorded in Brazil when compared with results in the state of Mato Grosso do Sul (91.6%), 4 Londrina, PR (49.2%), 12 Divinópolis, MG (49.5%), 13 Salvador, BA (51%), 14 Belo Horizonte, MG (56%), 15 northwest Paraná (59%), 16 São José do Rio Preto, SP (62%), 17 in the states of Tocantins (63%) 9 and Sergipe (69.3%), 7 and in Ilhéus, BA (72.3%). 8 In general, a metanalysis reported a seroprevalence of 61.2% in 27 pooled studies in Brazil. 18 International studies show that the global seroprevalence of IgG against *T. gondii* is 32.9% (95%CI: 29.4–36.4), but higher in the Americas, with a rate close to that found in the present study (45.2%, 95%CI: 33.4–53.4), and lower in the Western Pacific (11.2%, CI9 5%: 7.8–15.1). 19 However, a study conducted in Peru reported a lower seroprevalence of toxoplasmosis (35.8%). 20 Overall, seroprevalence depends on the specific community in which the data were collected. The collected values can be extremely different in the same country if the areas sampled have different health realities.

The issue has become more important due to the increased susceptibility of pregnant women and the current level of misinformation about toxoplasmosis considering that 73 patients reported they had never heard of toxoplasmosis and 81 women reported having heard of toxoplasmosis but did not know what it meant. Since 68.4% (154/225) of the patients in the studied group did not understand what toxoplasmosis infection meant and, therefore, would have difficulty carrying out prophylactic measures. Additional noteworthy data were found in a study conducted in Divinópolis, MG, 13 which reported that 93% of women had little or no knowledge of toxoplasmosis.

In the present study, elementary education or less was considered a risk factor for seropositivity to *T. gondii* , which could be due to a lack of infectious disease knowledge. Other studies associated less education with increased infection, as in Salvador, BA, 14 where it was observed that the higher the education level, the lower the prevalence of *T. gondii* infection (p = 0.01). This change was also observed in Londrina, PR, 12 where patients with complete or incomplete elementary education had a higher risk of toxoplasmosis infection (p < 0.001). The same association, however, was not found in Ilhéus, BA 8 (p = 0.106).

Another relevant factor in the present study was parity, in which primiparous was a protective factor. This may be related to the fact that most primiparous patients are younger. These younger patients have years of potential exposure ahead of them, due to the possibility of being infected in future pregnancies. However, a more complete analysis of the relationship between age and parity would be necessary to confirm this speculation. The same association was reported in Ilhéus, BA, 8 with primiparous women being protected against infection (p = 0.015, OR = 1.61, 95%CI 1.06–2.43).

Regarding patient age, the present study showed no statistically significant relationship between the different age groups analyzed (p = 0.0790), similar to findings from Ilhéus, BA 8 (p = 0.102). Other studies reported an association between age and infection rates. In Londrina, PR, 12 for example, there was a relationship between increasing age and the presence of IgG antibodies to *T. gondii* (p = 0.033), corroborating a study that included the entire northwest of Paraná 16 (p < 0.001).

The samples of this investigation were obtained in a reference tertiary hospital, that cares for pregnant women from 26 municipalities (approximately 2 million people), therefore allows us to consider that the generalization of this sample is one of the strengths of this research. Not evaluating eating habits with the necessary depth, especially in seropositive patients, can be considered one of the weaknesses of this research.

Thus, since toxoplasmosis is a preventable disease requiring prophylactic prevention measures, the lack of information assessed in this study demonstrates that the risk of contamination, given the high serological susceptibility, is relevant and should not be ignored by the public health system. Educational measures are necessary to help prevent acute maternal toxoplasmosis and the vertical transmission of *T. gondii* .

## Conclusion

The prevalence of toxoplasmosis was 40% in puerperal women, demonstrating that the risk of infection during pregnancy is high. Due to limited patient knowledge of toxoplasmosis infection and its forms of transmission, many patients do not take prophylactic measures to prevent infection. Seroprevalence was higher in postpartum women with low or no education. Thus, health education strategies are necessary to reduce acute maternal toxoplasmosis and the vertical transmission of *T. gondii.* Our study demonstrates the need to inform health bodies and prenatal care centers in Ribeirão Preto and the region about the importance of educating the vulnerable pregnant population about acute toxoplasmosis and the vertical transmission of the *Toxoplasma gondii* .

## References

[1] Arora N Sadovsky Y Dermody T S Coyne C B Microbial vertical transmission during human pregnancy Cell Host Microbe 2017 21 05 561 567 10.1016/j.chom.2017.04.007 28494237 PMC6148370

[2] Sarvi S Nayeri Chegeni T Sharif M Congenital toxoplasmosis among Iranian neonates: a systematic review and meta-analysis Epidemiol Health 2019 41 e2019021 10.4178/epih.e2019021 31096746 PMC6635660

[3] Peyron F L'ollivier C Mandelbrot L Maternal and congenital toxoplasmosis: diagnosis and treatment recommendations of a French Multidisciplinary Working Group Pathogens 2019 8 01 24 10.3390/pathogens8010024 30781652 PMC6470622

[4] Figueiró-Filho E A Lopes A H Senefonte F R Acute toxoplasmosis: study of frequency, rate of vertical transmission and the relation between maternal-fetal diagnostic tests in pregnant women in a state in the Midwest Region of Brazil Rev Bras Ginecol Obstet 2005 27 08 442 449 10.1590/S0100-72032005000800002

[5] Duarte G [Toxoplasmosis] Ribeirão Preto FUNPEC 2004 179 186. Portuguese.

[6] Inagaki A D Oliveira L A Oliveira M F [Seroprevalence of antibodies for toxoplasmosis, rubella, cytomegalovirus, syphilis and HIV among pregnant women in Sergipe] Rev Soc Bras Med Trop 2009 42 05 532 536 10.1590/s0037-86822009000500010Portuguese. 19967235

[7] Costa G B De Oliveira M C Gadelha S R Infectious diseases during pregnancy in Brazil: seroprevalence and risk factors J Infect Dev Ctries 2018 12 08 657 665 10.3855/jidc.9492 31958329

[8] Gontijo da Silva M Clare Vinaud M de Castro A M Prevalence of toxoplasmosis in pregnant women and vertical transmission of Toxoplasma gondii in patients from basic units of health from Gurupi, Tocantins, Brazil, from 2012 to 2014 PLoS One 2015 10 11 e0141700 10.1371/journal.pone.0141700 26558622 PMC4641701

[9] Martinez E Z [Biostatistics for undergraduate courses in the health area] São Paulo Blucher 2015. Portuguese.

[10] Ministério da Saúde, Instituto Sírio-Libanês de Ensino e Pesquisa Protocolos da atenção básica: saúde das mulheres Brasília, DF Ministério da Saúde 2016

[11] Harris P A Taylor R Thielke R Payne J Gonzalez N Conde J G Research electronic data capture (REDCap)--a metadata-driven methodology and workflow process for providing translational research informatics support J Biomed Inform 2009 42 02 377 381 10.1016/j.jbi.2008.08.010 18929686 PMC2700030

[12] Lopes F M Mitsuka-Breganó R Gonçalves D D Factors associated with seropositivity for anti-Toxoplasma gondii antibodies in pregnant women of Londrina, Paraná, Brazil Mem Inst Oswaldo Cruz 2009 104 02 378 382 10.1590/s0074-02762009000200036 19430668

[13] Fonseca A L Silva R A Fux B Madureira A P Sousa F F Margonari C Epidemiologic aspects of toxoplasmosis and evaluation of its seroprevalence in pregnant women Rev Soc Bras Med Trop 2012 45 03 357 364 10.1590/s0037-86822012000300015 22760136

[14] Avelar M V Martinez V O Moura D L Association between seroprevalence of IgG anti-Toxoplasma gondii and risk factors for infection among pregnant women in Climério de Oliveira Maternity, Salvador, Bahia, Brazil Rev Inst Med Trop São Paulo 2017 59 e90 10.1590/S1678-9946201759090 29267598 PMC5738996

[15] Carellos E V Andrade G M Aguiar R A [Evaluation of prenatal screening for toxoplasmosis in Belo Horizonte, Minas Gerais State, Brazil: a cross-sectional study of postpartum women in two maternity hospitals] Cad Saude Publica 2008 24 02 391 401 10.1590/s0102-311×2008000200018Portuguese. 18278286

[16] Ferezin R I Bertolini D A Demarchi I G [Prevalence of positive sorology for HIV, hepatitis B, toxoplasmosis and rubella in pregnant women from the northwestern region of the state of Paraná] Rev Bras Ginecol Obstet 2013 35 02 66 70 10.1590/s0100-72032013000200005Portuguese. 23412005

[17] Gonçalves M A Matos CdeC Spegiorin L C Oliani D C Oliani A H Mattos L C Seropositivity rates for toxoplasmosis, rubella, syphilis, cytomegalovirus, hepatitis and HIV among pregnant women receiving care at a public health service, São Paulo state, Brazil Braz J Infect Dis 2010 14 06 601 605 21340301

[18] Rostami A Riahi S M Gamble H R Global prevalence of latent toxoplasmosis in pregnant women: a systematic review and meta-analysis Clin Microbiol Infect 2020 26 06 673 683 10.1016/j.cmi.2020.01.008 31972316

[19] Bigna J J Tochie J N Tounouga D N Global, regional, and country seroprevalence of Toxoplasma gondii in pregnant women: a systematic review, modelling and meta-analysis Sci Rep 2020 10 01 12102 10.1038/s41598-020-69078-9 32694844 PMC7374101

[20] Silva-Díaz H Arriaga-Deza E V Failoc-Rojas V E Seroprevalence of toxoplasmosis in pregnant women and its associated factors among hospital and community populations in Lambayeque, Peru Rev Soc Bras Med Trop 2020 53 e20190164 10.1590/0037-8682-0164-2019 32187332 PMC7094055

